# Supplementary material for: Lead-free Zr-doped ceria ceramics with low permittivity displaying giant electrostriction
Source: Nat Commun. 2023 Nov 15;14:7371. doi: 10.1038/s41467-023-43032-5 (PMC10646075; doi:10.1038/s41467-023-43032-5)
Supplement: Supplementary file 1 — Supplementary Information [file 41467_2023_43032_MOESM1_ESM.pdf]

Supplementary Information

Lead-free Zr-doped ceria ceramics with low permittivity displaying giant electrostriction

Maxim Varenik†, Boyuan Xu† *et. al.*

†These authors contributed equally.

*Email: anatoly.frenkel@stonybrook.edu; yueqi@brown.edu; igor.lubomirsky@weizmann.ac.il

Supplementary Material

Table of contents

Page

1 **Supplementary Note 1: Crystal structure of $Zr_xCe_{1-x}O_2$ ceramics** 2
2 **Supplementary Note 2: Grain size distribution of dense ceramic pellets** 3
3 **Supplementary Note 3: Ultrasound pulse echo measurement of elastic moduli** ... 4
4 **Supplementary Note 4: SQUID magnetometry** 6
5 **Supplementary Note 5: Impedance Spectroscopy** 8
6 **Supplementary Note 6: Converse electrostriction strain coefficient.** 10
7 **Supplementary Note 7: X-ray absorption spectroscopy** 11
8 **Supplementary Note 8: DFT-based modelling of Zr doped ceria.** 14
9 **Supplementary Note 9: Real time electrostriction measurements** 21
10 **Supplementary Note 10: Oxidation treatment for 10 mol% Zr doped ceria** 22
11 **Supplementary Note 11: Electrostrictive relaxation in PMN-PT** 23

1 Supplementary Note 1: Crystal structure of $Zr_xCe_{1-x}O_2$ ceramics

Cubic lattice constants (**a**), determined under ambient conditions by X-ray diffraction (Rigaku, Ultima III), were observed to depend on dopant size relative to that of Ce. Local distortion of fluorite symmetry at low dopant concentration due, at least in part, to size mismatch of the cations, are not detected by XRD as they lack long range correlation. Shannon crystal radii (Å) for ligand coordination number 6-8: **Zr^{+4}** 0.86 - 0.98; **Ce^{+4}** 1.01-1.11.

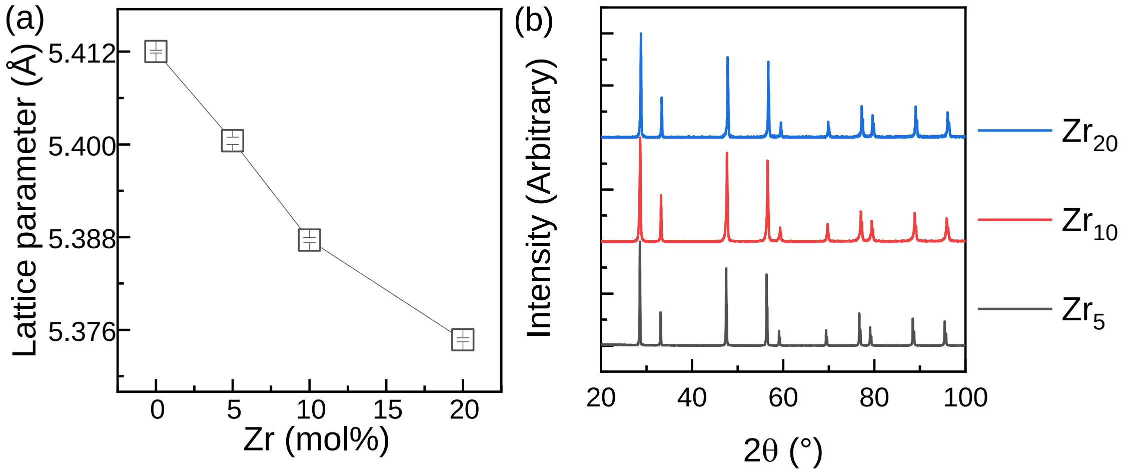

Supplementary Figure 1. (a) Lattice parameters of oxidized $Zr_xCe_{1-x}O_2$ ceramics were calculated by linear regression based on the indexing of 10 diffraction peaks according to $Fm\bar{3}m$ symmetry from (b). Prior to XRD measurement under ambient conditions, samples were heated at 773K for 5 h in pure oxygen atmosphere to compensate for possible oxygen loss during sintering. For most cases, the error bars are smaller than the size of the data symbol.

2 Supplementary Note 2: Grain size distribution of dense ceramic pellets

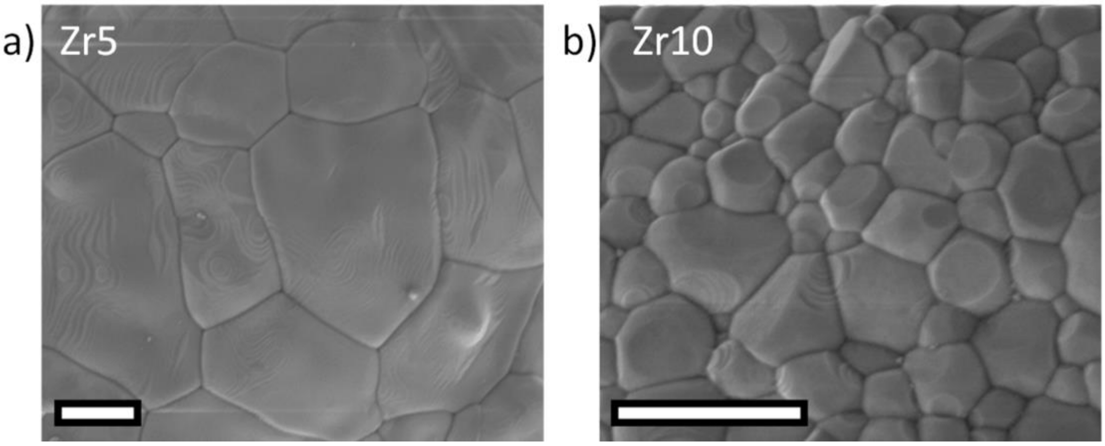

Supplementary Figure 2. SEM (Zeiss Sigma 500) micrographs of the circumferential surface of ceria ceramic pellets doped with: a) 5mol% Zr and b) 10mol% Zr. Prior to SEM measurements, samples were heated at 773K for 5 h in pure oxygen atmosphere to compensate for possible oxygen loss during sintering. Scale bars indicate 1μm.

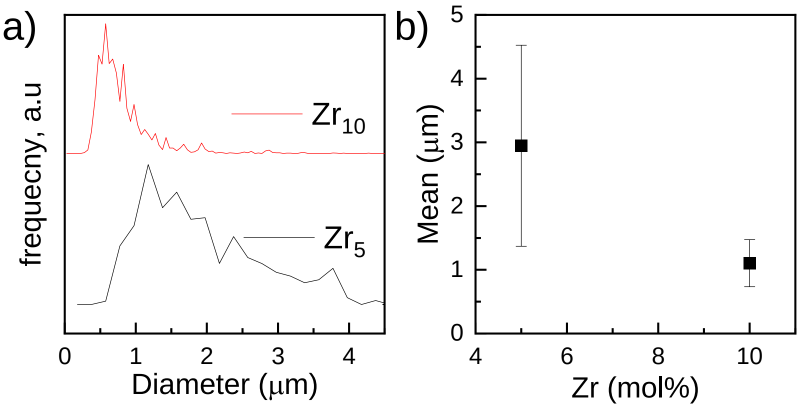

Supplementary Figure 3. a) Grain size distribution; and b) mean grain size determined by the lineal intercept method, including the x1.56 correction factor which accounts for grains which may be only partially visible at the imaged surface [1]. Error bars are standard deviation for >100 grains.

3 Supplementary Note 3: Ultrasound pulse echo measurement of elastic moduli

USTOF (ultrasound time of flight; ultrasound pulse echo) measurements. Shear (transverse, VS) and longitudinal, (VL) sound velocities were determined with accuracy better than 0.25% (pellet height measured with uncertainty ≤ 0.15%) with USTOF instrumentation and protocol as described in reference [2] and in previous reports.[3, 4, 5, 6] USTOF was measured using transducers coupled directly to the pellets with high viscosity commercial honey without external force. Correction for porosity $< 6\, vol\%$ was performed as described previously[3, 4]. Measurements were performed on oxidized pellets.

Shear (G_0 or C_{44}) and longitudinal (C_{11}) moduli were calculated from the corresponding sound velocities:

(Supplementary Eq. 1) $$C_{11} = \rho_m \cdot V_L^2 \text{ and } G_0 = \rho_m \cdot V_S^2,$$

where ρ_m is the measured pellet density. These moduli were used to calculate the Young's (Y_0) and bulk (B_0) moduli, and Poisson's ratio (υ_0):

(Supplementary Eq. 2) $$Y_0 = G_0 \frac{3V_L^2 - 4V_S^2}{V_L^2 - V_S^2},\ \upsilon_0 = \frac{Y_0}{2G_0} - 1,\ B_0 = \frac{Y_0 \cdot G_0}{3(3G_0 - Y_0)}.$$

To correct for porosity (p), the dynamic model developed by Ledbetter *et al.* [7, 8] leads to:

(Supplementary Eq. 3) $$G_D = \frac{-F + \sqrt{F^2 - 4AC}}{2A} \quad \text{and} \quad B_D = B_0 \frac{4G_D}{4(1-p)\cdot G_D - 3p \cdot B_0}\ ,$$

where $A = 8\frac{1-p}{3}$; $C = -3G_0 \cdot B_0(1+p)$; $F = (3-2p) \cdot B_0 - (8/3 + 4p) \cdot G_0$.

The subscript "0" denotes the values of the elastic moduli before correction for porosity, calculated using (Supplementary Eq. 1 and (Supplementary Eq. 2, while the subscript "D" denotes the values corrected for porosity according to the dynamic model. To reliably apply the correction, porosity must be less than 6vol%, as was indeed the case for the ceramic pellets studied here, verified by Archimedes method.

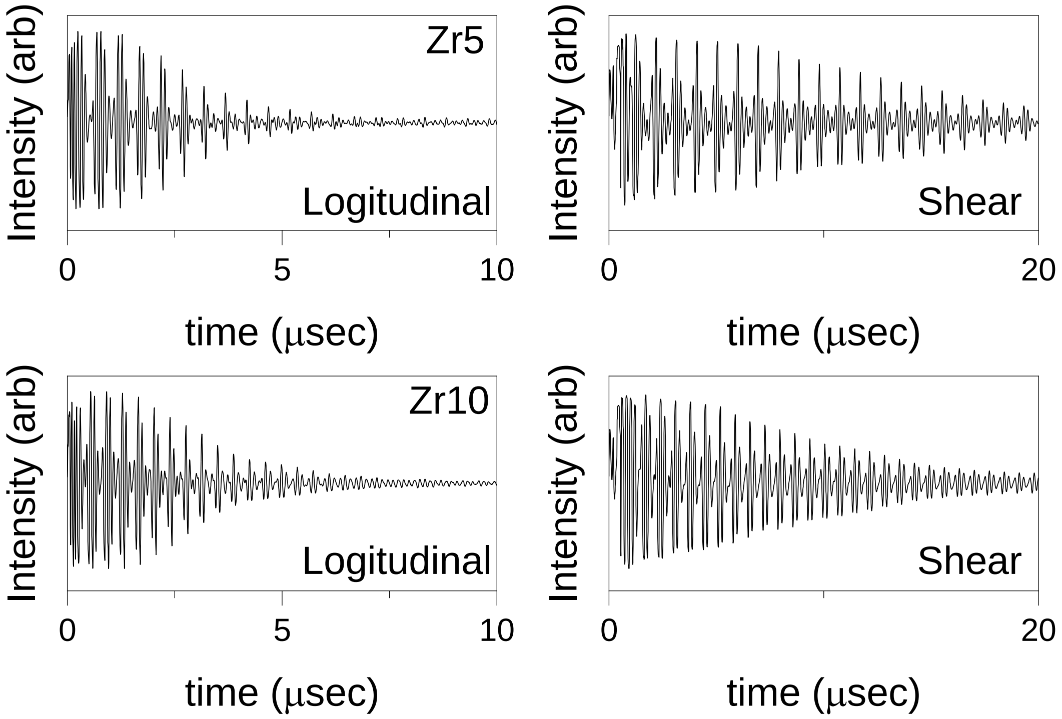

Supplementary Figure 4. Time decay of ultra-sound pulse echo (left – longitudinal, right – shear), of Zr_x-doped ceria: from top to bottom: x = 0.05, and 0.1.

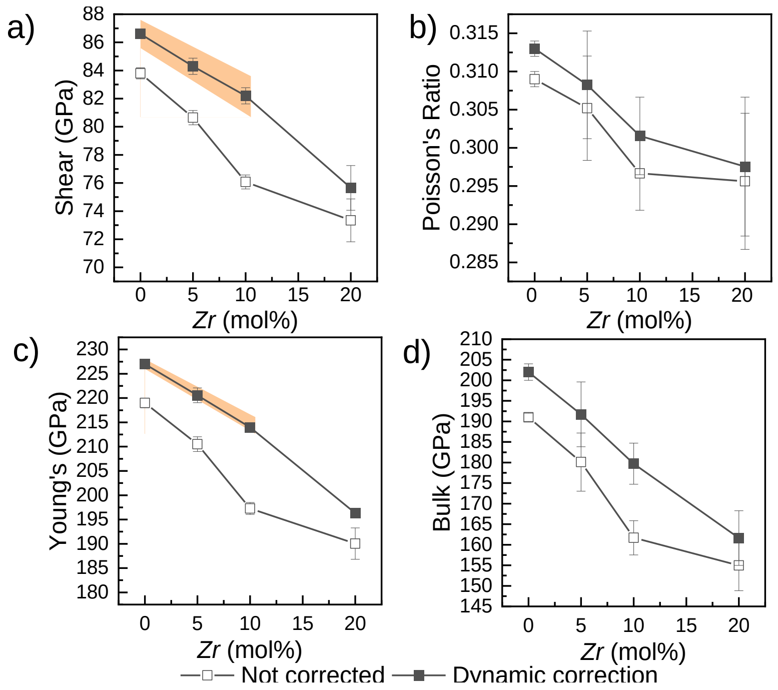

Supplementary Figure 5. Uncorrected and porosity-corrected (a) shear modulus, (b) Poisson's ratio, (c) Young's modulus, and (d) bulk modulus, calculated from ultrasound pulse echo measurements. Hollow symbols – uncorrected values; filled symbols – dynamic correction according to Ledbetter, [7, 8]. The highlighted region in (a), (c), and (d) is the predicted decrease according to [3, 4]. Measurements were performed in triplicate on each pellet.

4 Supplementary Note 4: SQUID magnetometry

The magnetization of the re-oxidized samples, $T = 2$ K (Supplementary Figure 6), saturates at significantly lower values than the reduced samples. Magnetization drops sharply following co-doping with La because the contribution of Ce^{3+} is suppressed.

a)

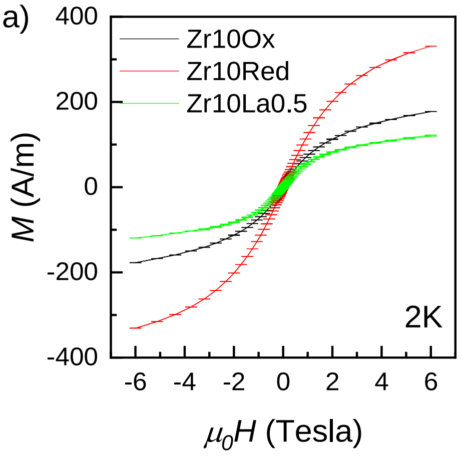

Supplementary Figure 6. Saturation magnetization measurements as measured at T = 2K in the SQUID magnetometer for fragments of 10mol% Zr doped ceria pellets. Measurements were made both before (Red - red curve), and following, re-oxidation (Ox-black curve).

The data in Supplementary Figure 6 cannot be fit to Langevin-type curves with physically reasonable parameters, likely because of the common presence of ~100ppm of magnetic impurities [9]. However, point by point calculation of difference magnetization curves (reduced minus re-oxidized Zr-doped ceramics) (Supplementary Figure 7) effectively cancels the contribution of the magnetic impurities; indeed, these can be fit to Langevin-type curves. The resulting fit parameters find approx. 500ppm Ce^{3+} ions for the reduced Zr samples while sample oxidation reduces this value to ~ 100 ppm. The latter value is comparable to known concentrations in undoped or aliovalent doped ceria under ambient conditions [9].

a)

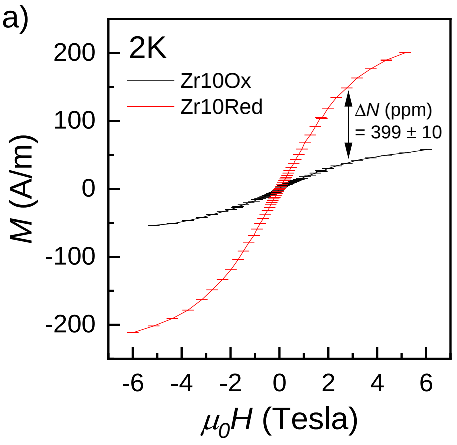

Supplementary Figure 7. Difference magnetization curves calculated with data presented in Supplementary Figure 6: magnetization of a fragment of a reduced (red curve) or oxidized (black curve) 10 mol% Zr doped ceria ceramic. Fitting parameters are listed in Supplementary Table 1.

Supplementary Table 1. Fitting parameters (Matlab®) for the Langevin equation (Eq. 2 in the main text) $M = Ng\mu_B J \cdot L(\eta) + \chi_0 \cdot H$, where N is the number of magnetic species per unit volume (m^{-3}); g is the Landé g-factor; μ_B is the Bohr magneton; $J = |L \pm S|$ and $L(\eta)$ is the Langevin function $L(\eta) = coth(\eta) - \frac{1}{\eta}$, where η is the ratio of the magnetic to thermal energy, $\eta = \frac{g\mu_0\mu_B J}{k_B T} \cdot H$; μ_0 is the vacuum magnetic permeability; k_B is the Boltzmann constant; T is absolute temperature (K); and H is the magnetic field strength (A m^{-1}), χ_0 is a temperature independent contribution which accounts for diamagnetic and Van Vleck susceptibility for curves in Supplementary Figure 7. The value of χ_0 was taken from previous studies[10].

	**x**	**N**	**gJ**	**R^2 adj**
**$M_{Reduced}$-$M_{Oxidized}$**	0.1	398±9	2.15±0.05	0.9999

5 Supplementary Note 5: Impedance Spectroscopy

Room temperature impedance spectroscopy measurements were conducted with a Novocontrol Alfa dielectric analyzer in high voltage mode. Applied voltage was: 10 V_{AC}, 1 MHz–1 mHz; 0 V_{DC}.

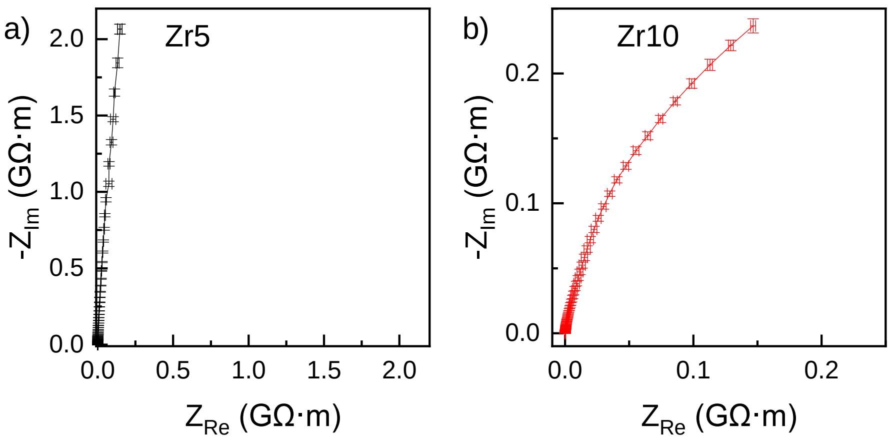

Supplementary Figure 8. Room temperature Nyquist plots for 5 mol% (a) and 10 mol% (b) Zr-doped ceria pellets: 0 V_{DC} bias, 10 V_{AC}, frequency range 1 MHz-1mHz, measured with upper spring-loaded electrode. Measurements were made in the same device as the converse electrostriction measurements.

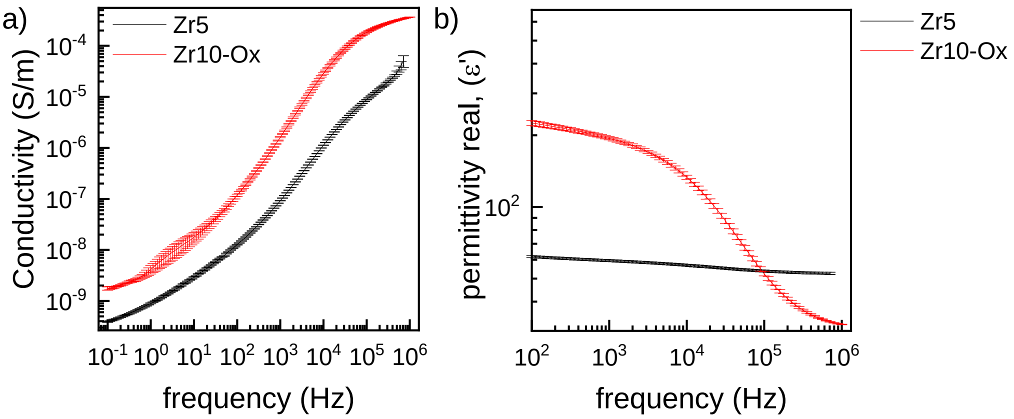

Supplementary Figure 9. Room temperature (a) conductivity and (b) real component of the relative dielectric permittivity for Zr-doped ceria pellets 0 V_{DC} bias, 10 V_{AC}, frequency range 1 MHz-100Hz, measured with stainless steel electrodes, the upper one being spring loaded. Measurements were made in the same device as the converse electrostriction measurements.

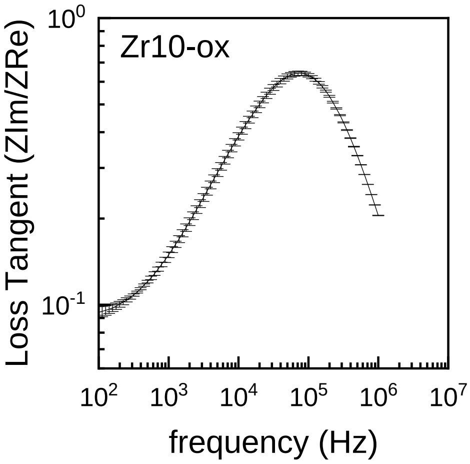

Supplementary Figure 10. Room temperature loss tangent for oxidized, 10mol%Zr-doped ceria pellets, 0 VDC bias, 10 VAC, frequency range 1 MHz-100Hz, measured with stainless steel electrodes of which the upper one was spring-loaded. Impedance measurements were made in the same device as the converse electrostriction measurements.

6 Supplementary Note 6: Converse electrostriction strain coefficient.

The converse electrostriction setup is pictured below (Supplementary Figure 11):

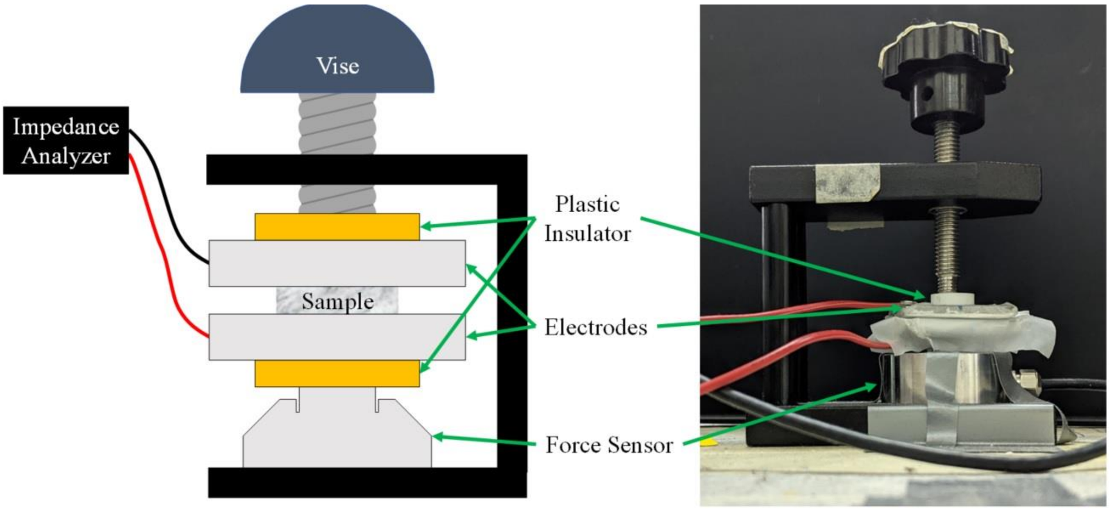

Supplementary Figure 11. Schematic of a converse electrostriction measurement apparatus in which force ⩽ 10 kg is applied uniaxially to the ceramic sample under ambient conditions.

The converse electrostriction strain coefficient is defined as:

(Supplementary Eq. 4) $$M = \varepsilon_0 \frac{\partial \varepsilon_r}{\partial s},$$

where ε_r is the relative permittivity of the ceramic measured using impedance spectroscopy; ε_0 is the permittivity of free space; s is the uniaxial compressive stress applied to the sample (a diagram of the screw vise is pictured below), calculated using a force sensor (MTI-XTL EQ-LC-KIT-5) and known sample dimensions. Rewriting Supplementary Eq. 4 gives the converse longitudinal electrostriction strain coefficient:

(Supplementary Eq. 5) $$\varepsilon_r = M \frac{s}{\varepsilon_0} + \varepsilon_r|_{s=0}.$$

Supplementary Figure 12, indeed confirms the linear relationship.

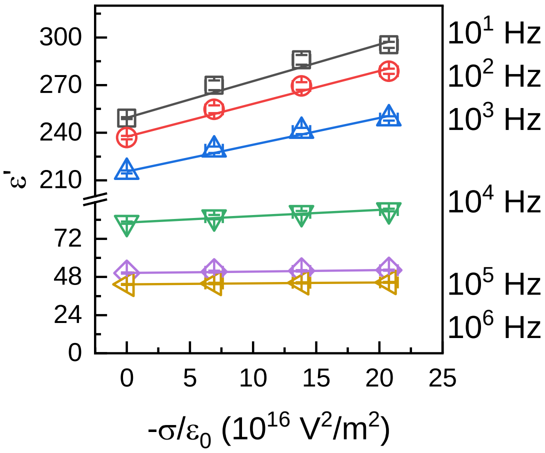

Supplementary Figure 12. Increase in the relative real component of the dielectric permittivity ε_{Real} under uniaxial compressive stress for an oxidized 10 mol% Zr-doped ceria ceramic pellet.

7 Supplementary Note 7: X-ray absorption spectroscopy

Zr K-edge XANES data, presented in Supplementary Figure 13, demonstrate that the oxidation states of Zr in all samples are close to that in ZrO_2 (Zr^{4+}). However, data for Zr doped ceria samples show clear differences in the white line region from the ZrO_2 standard (monoclinic under ambient conditions) that has a single peak (Figure 5a main text, inset). The double-peak structure is indicative of a locally cubic ZrO_2 environment in Zr-doped ceria for all dopant concentrations studied here and extent of reduction. Taken together, XRD, XANES and EXAFS point to a substitutional replacement of Zr in the (cubic) fluorite structure of CeO_2.

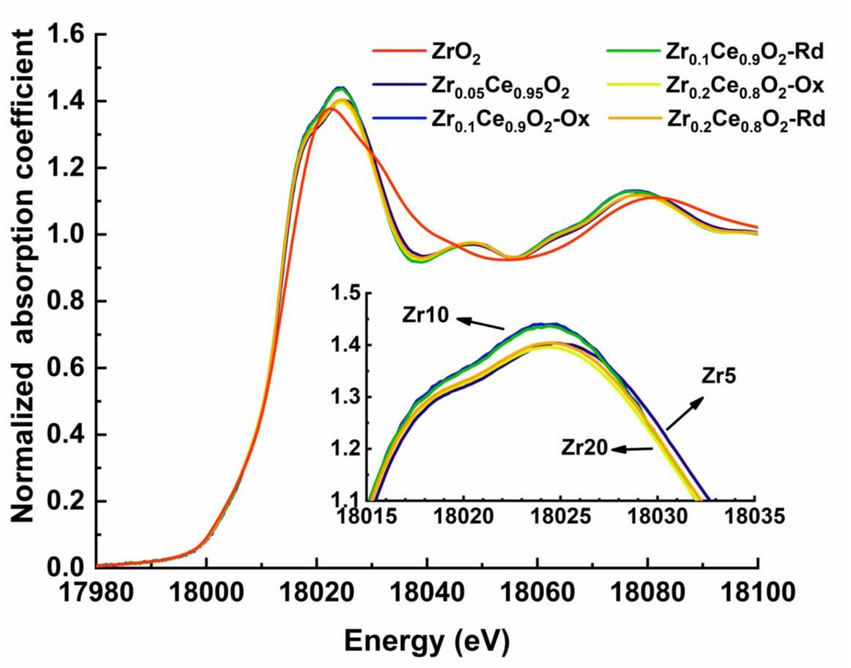

Supplementary Figure 13. The normalized Zr K-edge XANES spectrum of Zr-doped ceria. For comparison, the spectrum of ZrO_2 was also included. Inset: Zr5, Zr10, and Zr20 refer to 5, 10 or 20mol% Zr-doped ceria, respectively.

EXAFS analysis was performed using the Demeter package [11]. Zr foil was first measured on the same beamline and was analyzed to obtain the passive electron reduction factor (S_0^2). Supplementary Table 2 contains a summary of the best fit results for Zr foil. Data and theoretical fits for Zr K-edge EXAFS spectra are presented in k-space and r-space, as shown in Supplementary Figure 14. S_0^2 (0.99) was used in fitting the spectra of Zr-doped ceria. The nearest neighbor anion coordination number (CN) for Zr in Zr doped ceria samples was set as 8. Supplementary Table 3 contains the summary of the best fit results for all data. The data and theoretical fits for Zr K-edge EXAFS fitting are presented in k-space and r-space, as shown in Supplementary Figure 15.

Supplementary Table 2. The best-fitting results for Zr foil.

Sample	Path	CN	S_0^2	R (Å)	σ^2 (Å^2)
**Zr foil**	Zr-Zr	12	0.99±0.07	3.200±0.003	0.0096±0.0004

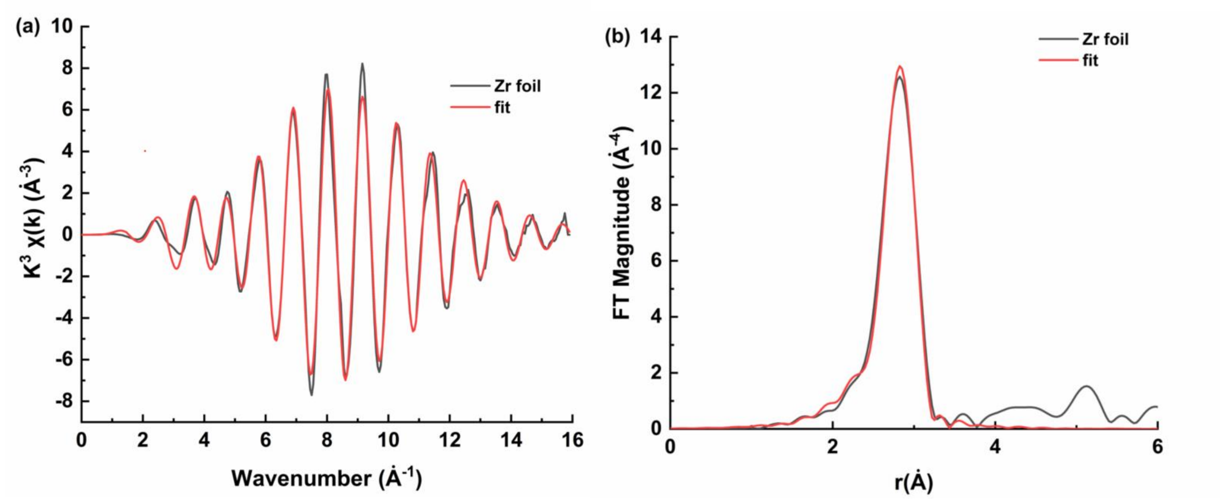

Supplementary Figure 14. Comparison between experimental and fitted spectra for Zr foil are shown in k-space (a) and, following Fourier transformation of the k range 2-14.5 Å^{-1}, also in r-space (b).

Supplementary Table 3. Zr K-edge EXAFS best fit parameters (coordination numbers, CN, bond lengths, R, and variance, σ^2) for all samples.

Samples	Path	CN	R (Å)	σ^2 (Å^2)
**$Zr_{0.05}Ce_{0.95}O_2$**	Zr-O	8	2.20±0.01	0.0100±0.0014
	Zr-Ce	12	3.78±0.01	0.0077±0.0004
**$Zr_{0.1}Ce_{0.9}O_2$-Ox**	Zr-O	8	2.23±0.01	0.0090±0.0014
	Zr-Ce	12	3.79±0.01	0.0062±0.0003
**$Zr_{0.1}Ce_{0.9}O_2$-Rd**	Zr-O	8	2.22±0.01	0.0093±0.0014
	Zr-Ce	12	3.79±0.01	0.0063±0.0003
**$Zr_{0.2}Ce_{0.8}O_2$-Ox**	Zr-O	8	2.21±0.02	0.0107±0.0018
	Zr-Ce	12	3.79±0.01	0.0073±0.0004
**$Zr_{0.2}Ce_{0.8}O_2$-Rd**	Zr-O	8	2.21±0.01	0.0105±0.0016
	Zr-Ce	12	3.78±0.01	0.0077±0.0004

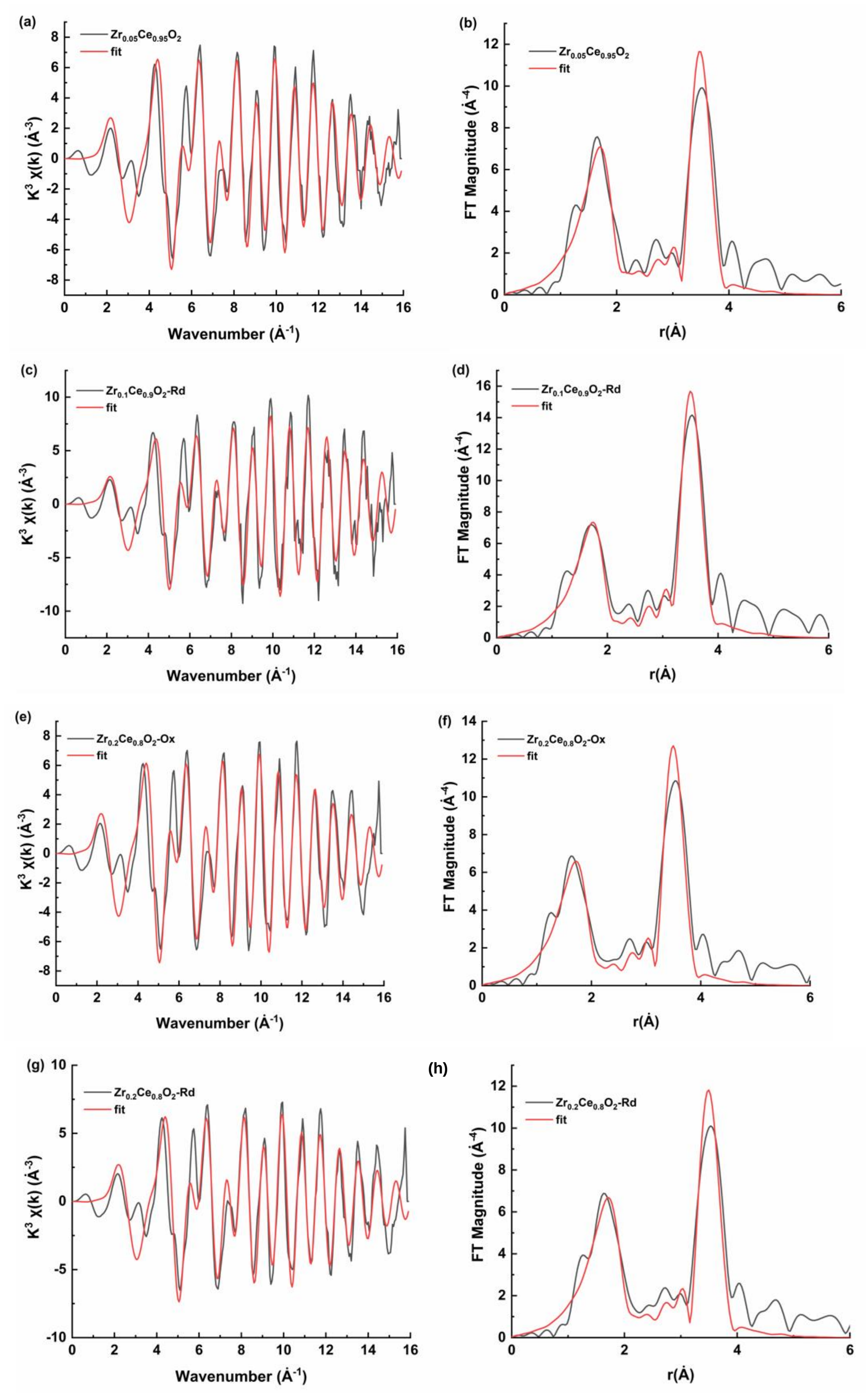

Supplementary Figure 15. Measured and fitted EXAFS spectra for $Zr_{0.05}Ce_{0.95}O_2$, $Zr_{0.1}Ce_{0.9}O_2$-Rd, $Zr_{0.2}Ce_{0.8}O_2$-Ox, and $Zr_{0.2}Ce_{0.8}O_2$-Rd. Panels a, c, e, g: k space; panels b, d, f, h: r space. The k range for the Fourier transform was 2-14.5 $Å^{-1.}$ Ox- fully oxidized powder; Rd-reduced powder. 5mol% Zr doped ceria showed no evidence of reduction during sintering.

8 Supplementary Note 8: DFT-based modelling of Zr doped ceria.

Subsection 8.1 **DFT calculations**

DFT calculations are performed using the Projector Augmented Wave (PAW) method [12] implemented in the Vienna Ab initio Simulation Package (VASP). The generalized gradient approximation of Perdew, Burke, and Ernzerhof (PBE)[13] was used for the DFT exchange correlation functional. Hubbard-U correction for Ce 4f orbitals was chosen to be $U_{\mathrm{eff}} = 4.5$ eV, as proposed by Dudarev et al.[14]. The plane-wave cutoff energy was set to 500 eV with $2 \times 2 \times 2$ Gamma point Monkhorst-Pack k-point meshes for a $2 \times 2 \times 2$ 96-atom supercell. The electronic and atomic relaxation convergence criteria were 1×10^{-6} and 0.01 eV Å^{-1}, respectively. For 300K AIMD (*ab initio* molecular dynamics) calculations, the time step is set to be 1 fs with total energy evaluated at an accuracy of 1×10^{-4} eV per atom.

Subsection 8.2 **Chemical expansion coefficient tensor**

The chemical expansion coefficient tensor was computed following the method derived by Gillian [15] and others[16, 17] and procedures provided by Das [18]. The main idea is that at equilibrium, energy changes due to substitution-induced local lattice distortion are balanced by the long-range elastic energy. Zr substitutional defect formation energy at a strain state $\boldsymbol{u}$, $E^{f}_{Zr,u}$ was defined as[19]

(Supplementary Eq. 6) $$E^{f}_{Zr,u} = E^{Zr}_{CeO_2,u} - E^{s}_{CeO_2,u} - (E_{ZrO_2} - E_{CeO_2}),$$

where $E^{Zr}_{CeO_2,u}$ and $E^{s}_{CeO_2,u}$ are the total energies of the fully relaxed Zr-doped CeO_2 and pure CeO_2 with the same supercell size at a different strain state $\boldsymbol{u}$, respectively. E_{CeO_2} and E_{ZrO_2} are the bulk energies for one formula unit of CeO_2 (-24.488eV) and ZrO_2, (-28.56eV), respectively. The short-range elastic dipole tensor $\boldsymbol{G}$ can be associated with $E^{f}_{Zr,u}$ at a given applied strain tensor through a first order Taylor expansion:

(Supplementary Eq. 7) $$E^{f}_{Zr,u} = E^{f}_{Zr,u=0} + \boldsymbol{G}:\boldsymbol{u}.$$

Calculating $E^{f}_{Zr,u}$ at different strain states along different strain paths, followed by linear fitting, provides each component of the $\boldsymbol{G}$ tensor. For Zr off-centered structures, non-fully relaxed structures were used. The starting structures at zero strain were taken either from the *ab initio* Molecular Dynamics 300K (AIMD) 1 fs snapshots or ZrO_8-off centered structure with cations fixed. When strain $\boldsymbol{u}$ is applied, the structures were deformed but the ions are not relaxed. Combining the elastic dipole tensor with the elastic stiffness tensor, $\mathbb{C}$, we obtain the dopant-induced strain tensor per Zr , $\boldsymbol{\alpha_C}$.

(Supplementary Eq. 8) $$\boldsymbol{\alpha}_C = -\frac{(\mathbb{C}^{-1}\boldsymbol{G})}{V_U},$$

where $V_U = 41.50$ Å^3 is the volume per formula unit of CeO_2 and $\mathbb{C}$ ($C_{11} = 343$ GPa, $C_{22} = 103$ GPa, $C_{44} = 54$ GPa) is the elastic stiffness tensor of CeO_2. The chemical strain, $\boldsymbol{u}_C$, for Zr concentration c_{zr}, can be obtained by

(Supplementary Eq. 9) $\boldsymbol{u}_C = \boldsymbol{\alpha}_C c_{Zr}$.

Subsection 8.3 **Cubic distortion parameters**

MO_8 cubic distortion analysis is implemented in analogy to MO_6 octahedral distortion analysis[20, 21], which depends on cubic distortion σ^2:

(Supplementary Eq. 10) $\sigma^2 = \frac{1}{8}\sum_{i=1}^{8}\left(l_i - l_{avg}\right)^2$,

where l_i and l_{avg} are individual M-O bond lengths and the averaged bond length in MO_8, respectively. The bond angle variance θ^2 is:

(Supplementary Eq. 11) $\theta^2 = \frac{1}{11}\sum_{i=1}^{12}(\theta_{O-Zr-O} - \theta_0)^2$.

here θ_{O-Zr-O} is the closest O-Zr-O bond angle; $\theta_0 \approx 70.528°$ is the bond angle in a perfect lattice.

Subsection 8.4 **Supercell electric dipole moment**

The overall supercell electric dipole moment can be calculated by taking into consideration all charges and positions of the 96 atoms:

(Supplementary Eq. 12) $\vec{\mu} = \sum_{i=1}^{N} q_i\vec{r}_i$,

where q_i is the charge of atom i, and $\vec{r}_i$ is the vector pointing to this atom from the origin, while the image atoms are considered due to the periodic boundary conditions. The electric dipole unit is selected to be debye (D), in CGS units, and 1 debye is approximately 0.2082 $e \cdot$ Å.

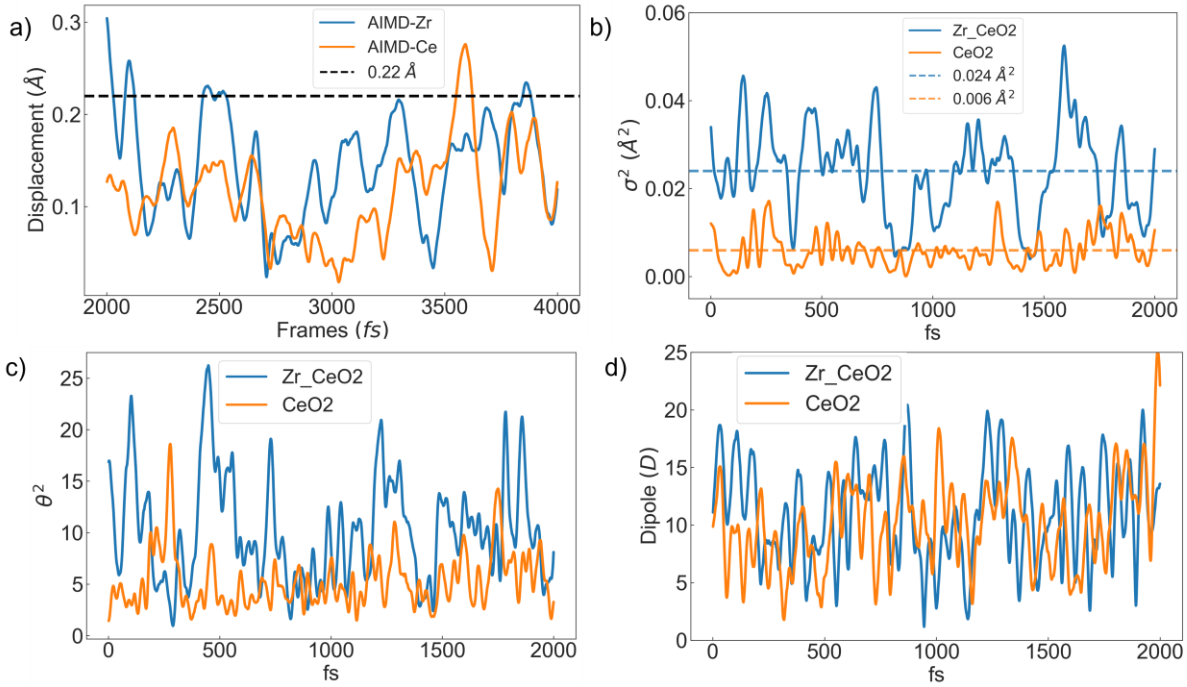

Supplementary Figure 16. a) The root mean square deviation (RMSD) of Zr and Ce displacement in the last 2 ps trajectories. The average displacement is 0.143 Å and 0.116 Å for Zr and Ce respectively. 0.22 Å is marked (- - - -) as the value that represents maximum Zr and Ce off-equilibrium displacements. b, c). The last 2 ps [MO_8] (M=Zr or Ce) cube distortion parameters of M-O bond lengths, σ^2 and O-M-O bond angles θ^2 (in degree^2) are plotted with respect to time. Time averaged values show ${\sigma^2}_{Zr}/{\sigma^2}_{Ce} = 0.024/0.006 \approx 4.000$, $\theta^2_{Zr}/\theta^2_{Ce} = 10.139/5.306 \approx 1.911$. d). The supercell electric dipole moment μ magnitude evolution with time. The electric dipole unit is debye (D), where $1D \approx 0.2082\ e \cdot$ Å. Time averaged values show $\mu_{Zr-CeO_2}/\mu_{CeO_2} = 10.944/10.046 \approx 1.089$.

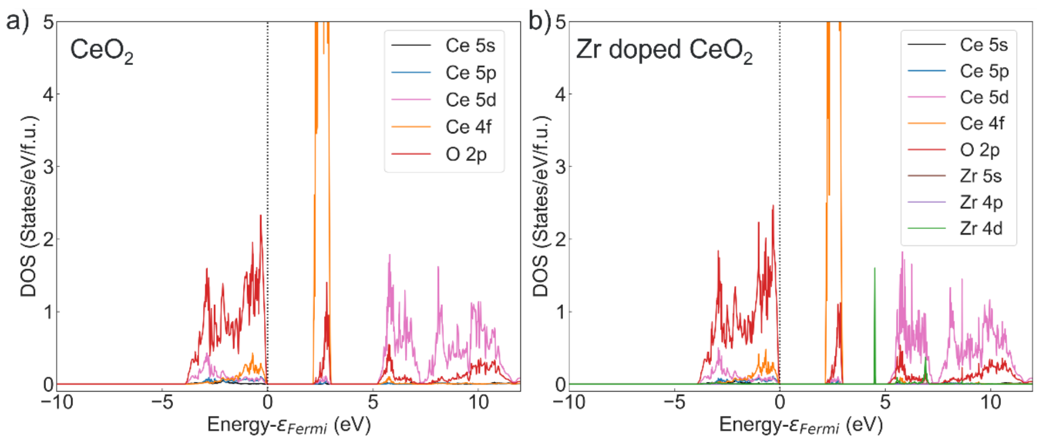

Supplementary Figure 17. The computed partial density of states (PDOS) are shown for (a) bulk CeO2 and (b) 3.125% Zr doped CeO2 with respect to the fermi level, which coexists with the Valence Band Maximum. Substitute Ce with Zr does not significantly alter its electronic properties, similar to those reported in the literature [21,22,23]. The experimentally measured band gap is predominantly O-2p with respect to conduction band Ce-5d, which stays 5.17 eV [24]. The energy difference between the occupied O-2p bands (in range [-3.93, 0] eV) and the unoccupied Ce-4f band (in range [2.16, 3.03] eV) stays at around 2.16 eV after Zr-doping. A new state belonging to unoccupied Zr-4d orbital appears at ~4.50 eV above the Fermi-level. In bulk ZrO_2, the conduction band is mainly derived from Zr 4d orbitals. This localized peak will have limited contributions to electron conduction but leading to a reduced band gap and increase the dielectric constant locally, which is inversely proportional to the band gap [25].

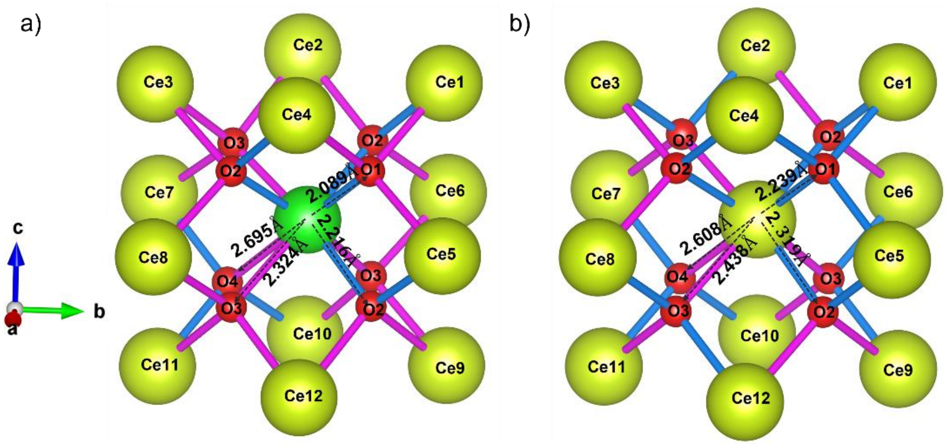

Supplementary Figure 18. Distorted MO_8Ce_{12} structure, M=Zr **(a)** or Ce **(b),** with labeled Ce atoms and numerical M-O bond lengths. M-O bonds fall into 4 groups (O1 to O4, red color) based on the extent of distortion. The M-O bond lengths in each group are almost the same. M-O bonds are colored pink (expansion) or blue (contraction).

Supplementary Table 4. Second nearest neighbor (NNN) Ce-O bond lengths, which fall into 4 groups, for selected 0.22 Å [111] displaced MO_8Ce_{12} structures (M=Zr or Ce). Reference bond length is 2.380Å. *i.e.*, Ce-O bond in fluorite CeO_2. See Supplementary Figure 18 for Ce label definitions.

O position	Group	Ce label	Ce-center	Zr-center
			Bond length Difference Å	Bond length Difference Å
[111]	1	Ce1	-0.026	0.001
		Ce4	-0.026	0.001
		Ce5	-0.024	0.002
$[\bar{1}11]$	2	Ce1	-0.054	-0.038
		Ce2	0.024	0.035
		Ce6	0.023	0.034
$[11\bar{1}]$	2	Ce5	-0.054	-0.038
		Ce9	0.023	0.034
		Ce12	0.023	0.034
$[1\bar{1}1]$	2	Ce3	0.024	0.035
		Ce4	-0.054	-0.038
		Ce8	0.023	0.034
$[\bar{1}\bar{1}1]$	3	Ce2	-0.013	0.026
		Ce3	-0.013	0.026
		Ce7	0.045	0.021
$[\bar{1}1\bar{1}]$	3	Ce6	-0.013	0.026
		Ce9	-0.012	0.028
		Ce10	0.046	0.023
$[1\bar{1}\bar{1}]$	3	Ce8	-0.013	0.026
		Ce11	0.046	0.023
		Ce12	-0.012	0.028
$[\bar{1}\bar{1}\bar{1}]$	4	Ce7	-0.002	-0.052
		Ce10	-0.004	-0.053
		Ce11	-0.003	-0.053

Supplementary Table 5. Fitted quadratic function ($ax^2 + bx + c + O(d)$) for different displacement conditions: Zr in [ZrO_8], Ce in [CeO_8], Ce near [ZrO_8].

Condition	Stiffness constant ($eV/Å^2$)	1st order coefficient ($eV/Å$)	Constant (eV)	Error (eV)
[100] Zr in O8	3.01038	0.00375	-787.24370	0.00092
[110] Zr in O8	2.59349	0.00459	-787.24003	0.00008
[111] Zr in O8	2.47217	0.00256	-787.23877	0.00004
[100] Ce in O8	6.13796	0.00068	-783.62846	0.00001
[110] Ce in O8	6.04633	0.00136	-783.62768	0.00005
[111] Ce in O8	6.06002	0.00042	-783.62800	0.00002
[100] Ce near Zr	5.96863	-0.00204	-787.23872	0.00001
[110] Ce near Zr	5.52310	0.01744	-787.23783	0.00026
[111] Ce near Zr	5.40348	-0.04438	-787.23858	0.00013

Supplementary Table 6. The computed elastic dipole tensor $\boldsymbol{G}$ and the dopant-induced strain tensor per Zr ion, $\boldsymbol{\alpha_C}$, for different Zr structures. The diagonalization operation gives 3 eigenvectors for each diagonalized matrix, they are the principal directions that the strain tensor $\boldsymbol{\alpha_C}$ is projected onto.

	$\boldsymbol{G}$	$\boldsymbol{\alpha_C}$		
	in Cartesian coordinates	in Cartesian coordinates	Diagonalized	Eigenvectors
Fully relaxed Zr at the Ce-center	$\begin{bmatrix} 9.358 & -0.001 & -0.001 \\ -0.001 & 9.358 & -0.001 \\ -0.001 & -0.001 & 9.358 \end{bmatrix}$	$\begin{bmatrix} -0.066 & 0 & 0 \\ 0 & -0.066 & 0 \\ 0 & 0 & -0.066 \end{bmatrix}$	$\begin{bmatrix} -0.066 & 0 & 0 \\ 0 & -0.066 & 0 \\ 0 & 0 & -0.066 \end{bmatrix}$	$[1 \quad 0 \quad 0]$ $[0 \quad 1 \quad 0]$ $[0 \quad 0 \quad 1]$
Snapshot from AIMD 300K 3500fs	$\begin{bmatrix} -3.539 & -0.089 & -0.089 \\ -0.089 & -3.154 & 1.017 \\ -0.089 & 1.017 & -3.038 \end{bmatrix}$	$\begin{bmatrix} 0.028 & 0.006 & 0.064 \\ 0.006 & 0.021 & -0.073 \\ 0.064 & -0.073 & 0.019 \end{bmatrix}$	$\begin{bmatrix} -0.078 & 0 & 0 \\ 0 & 0.031 & 0 \\ 0 & 0 & 0.115 \end{bmatrix}$	$[0.457 \quad -0.544, \quad -0.704]$ $[-0.750 \quad -0.661 \quad 0.024]$ $[0.478 \quad -0.517 \quad 0.710]$
Snapshot from AIMD 300K 3900fs	$\begin{bmatrix} -5.712 & -1.308 & 0.474 \\ 1.308 & -5.479 & 1.806 \\ 0.474 & 1.806 & -5.276 \end{bmatrix}$	$\begin{bmatrix} 0.042 & 0.093 & -0.034 \\ 0.093 & 0.038 & -0.129 \\ -0.034 & -0.129 & 0.035 \end{bmatrix}$	$\begin{bmatrix} 0.214 & 0 & 0 \\ 0 & 0.008 & 0 \\ 0 & 0 & -0.107 \end{bmatrix}$	$[-0.475 \quad -0.670 \quad 0.571]$ $[-0.817 \quad 0.094 \quad -0.569]$ $[-0.328 \quad 0.737 \quad 0.592]$
Zr-off center 0.22 Å along [111] direction	$\begin{bmatrix} 7.645 & -0.938 & -0.925 \\ -0.938 & 7.645 & -0.925 \\ -0.925 & -0.925 & 7.640 \end{bmatrix}$	$\begin{bmatrix} -0.054 & 0.067 & 0.066 \\ 0.067 & -0.054 & 0.066 \\ 0.066 & 0.066 & -0.054 \end{bmatrix}$	$\begin{bmatrix} 0.079 & 0 & 0 \\ 0 & -0.121 & 0 \\ 0 & 0 & -0.119 \end{bmatrix}$	$[-0.578 \quad -0.578 \quad -0.576]$ $[-0.696 \quad 0.718 \quad -0.022]$ $[-0.426 \quad -0.388 \quad 0.817]$

9 Supplementary Note 9: Real time electrostriction measurements

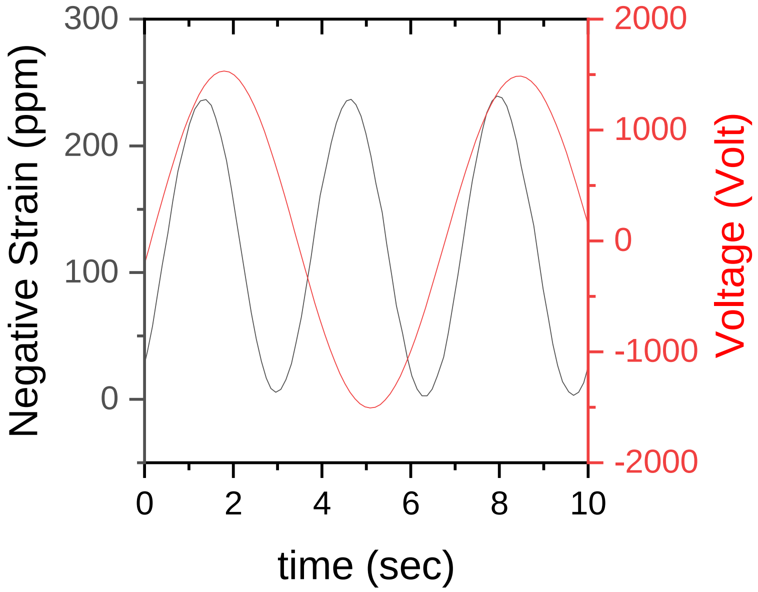

Supplementary Figure 19. Real time strain and voltage measurements during an electrostriction experiment at f = 0.15 Hz, V = 1550 V, 10 mol% Zr-doped (oxidized) ceria pellet. The phase lag does not represent an actual delay between the strain and applied voltage. The phase lag is introduced between the amplifier voltage monitor port, and the output of the capacitance meter. Both ports are connected to a Keithley 2000 digital multimeter from which data are captured by a VISA interface. The apparent phase lag is a result of the phase shift introduced by the presence of the multimeter, device interrogation sequence, and delay between the interrogation sequence and the measurements.

10 Supplementary Note 10: Oxidation treatment for 10 mol% Zr doped ceria

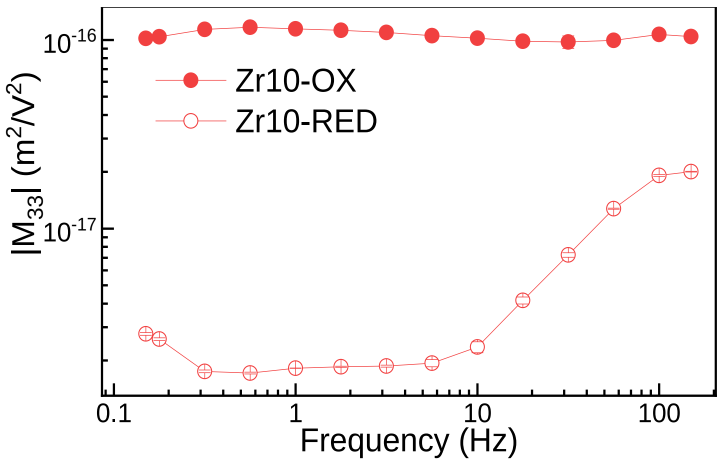

Supplementary Figure 20. Frequency dependence of the direct, longitudinal electrostriction coefficient $|M_{33}|$ of sintered ceria pellets doped with Zr^{4+}, both before (Red) and after (Ox) re-oxidation; Measurements were made in triplicate under ambient conditions; in some cases, error bars are smaller than the symbols.

11 Supplementary Note 11: Electrostrictive relaxation in PMN-PT

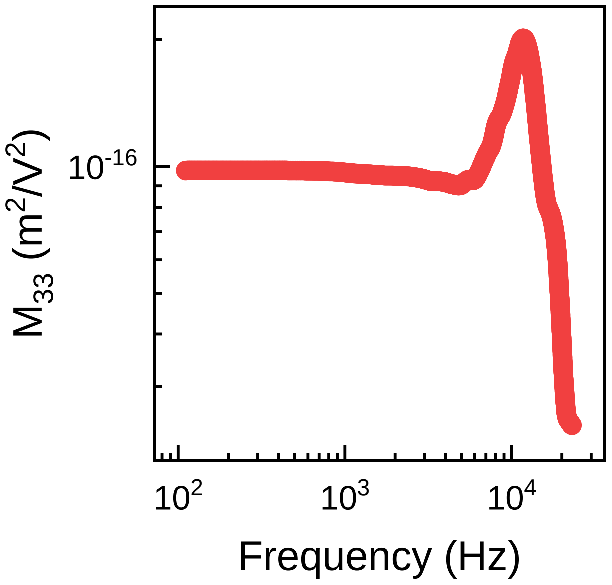

Supplementary Figure 21. Electrostriction strain coefficient in PMN-PT15 (TRS ceramics), at 0.2 kV/cm. A 50% reduction is observed at 19.4kHz. Thermal expansion is detected close to relaxation due to the increase in the imaginary component of the dielectric permittivity (ε''), thereby causing Joule heating. Below the relaxation frequency, the real component of the dielectric permittivity exceeds 10,000, which presents serious practical difficulties.

Supplementary References

1. Mendelson M. I., Average grain size in polycrystalline ceramics. J. Am. Ceram. Soc. **52**, 443-446 (1969).

2. Yeheskel O., Tevet O., Elastic moduli of transparent yttria. J. Am. Ceram. Soc. **82**, 136-144 (1999).

3. Varenik M., *et al.*, Oxygen vacancy ordering and viscoelastic mechanical properties of doped ceria ceramics. Scr. Mater. **163**, 19-23 (2019).

4. Yavo N., *et al.*, Elastic moduli of pure and gadolinium doped ceria revisited: sound velocity measurements. Scr. Mater. **123**, 86-89 (2016).

5. Hoedl M., *et al.*, Impact of point defects on the elastic properties of BaZrO3: Comprehensive insight from experiments and ab initio calculations. Acta Mater. **160**, 247-256 (2018).

6. Makagon E., Merkle R., Maier J., Lubomirsky I., Influence of hydration and dopant ionic radius on the elastic properties of BaZrO3. Solid State Ionics **344**, 115130 (2020).

7. Ledbetter H. M., Austin M. W., Kim S. A., Lei M., Elastic constants and Debye temperature of polycrystalline Y1Ba2Cu3O7−x. J. Mater. Res. **2**, 786-789 (2011).

8. Ledbetter H. M., Datta S. K., Effective Wave Speeds in an Sic-Particle-Reinforced Al Composite. J. Acoust. Soc. Am. **79**, 239-248 (1986).

9. Mogensen M., Sammes N. M., Tompsett G. A., Physical, chemical and electrochemical properties of pure and doped ceria. Solid State Ionics **129**, 63-94 (2000).

10. Varenik M., *et al.*, Van Vleck paramagnetism in undoped and Lu-doped bulk ceria. Phys. Chem. Chem. Phys. **20**, 27019-27024 (2018).

11. Ravel B., Newville M., ATHENA, ARTEMIS, HEPHAESTUS: data analysis for X-ray absorption spectroscopy using IFEFFIT. J. Synchrotron Radiat. **12**, 537-541 (2005).

12. Kresse G., Joubert D., From ultrasoft pseudopotentials to the projector augmented-wave method. Phys. Rev. B **59**, 1758-1775 (1999).

13. Perdew J. P., Burke K., Ernzerhof M., Generalized gradient approximation made simple. Phys. Rev. Lett. **77**, 3865-3868 (1996).

14. Dudarev S. L., Botton G. A., Savrasov S. Y., Humphreys C. J., Sutton A. P., Electron-energy-loss spectra and the structural stability of nickel oxide: An LSDA+U study. Phys. Rev. B **57**, 1505-1509 (1998).

15. Leslie M., Gillan M. J., The Energy and Elastic Dipole Tensor of Defects in Ionic-Crystals Calculated by the Supercell Method. J. Phys. C: Solid State Phys. **18**, 973-982 (1985).

16. Er D., *et al.*, A Model to Determine the Chemical Expansion in Non-Stoichiometric Oxides Based on the Elastic Force Dipole. J Electrochem Soc **161**, F3060-F3064 (2014).

17. James C., Wu Y., Sheldon B., Gil Y., Computational Analysis of Coupled Anisotropic Chemical Expansion in Li2-XMnO3-delta. Mrs Adv **1**, 1037-1042 (2016).

18. Das T., Nicholas J. D., Qi Y., Long-range charge transfer and oxygen vacancy interactions in strontium ferrite. J. Mater. Chem. A **5**, 4493-4506 (2017).

19. Sharma V., Kumar P., Dev P., Pilania G., Machine learning substitutional defect formation energies in ABO(3) perovskites. J. Appl. Phys. **128**, (2020).

20. Shannon R. D., Revised Effective Ionic-Radii and Systematic Studies of Interatomic Distances in Halides and Chalcogenides. Acta Crystallographica Section A **32**, 751-767 (1976).

21. Zhang X. H., *et al.*, FeO6 Octahedral Distortion Activates Lattice Oxygen in Perovskite Ferrite for Methane Partial Oxidation Coupled with CO2 Splitting. J Am Chem Soc **142**, 11540-11549 (2020).
